# LncRNA XIST facilitates hypertrophy of ligamentum flavum by activating VEGFA-mediated autophagy through sponging miR-302b-3p

**DOI:** 10.1186/s13062-023-00383-9

**Published:** 2023-05-24

**Authors:** Yanlin Cao, Jianjun Li, Sujun Qiu, Songjia Ni, Yang Duan

**Affiliations:** 1 Department of Spine Surgery, Zhujiang Hospital, Southern Medical University, Guangzhou, China; 2 Department of Orthopaedic Trauma, Zhujiang Hospital, Southern Medical University, Guangzhou, China

**Keywords:** Hypertrophy of ligamentum flavum, Long non-coding RNA, LncRNA XIST, miR-302b-3p, VEGFA, Autophagy

## Abstract

**Background:**

Increasing evidences have shown that long non-coding RNAs (lncRNAs) display crucial regulatory roles in the occurrence and development of numerous diseases. However, the function and underlying mechanisms of lncRNAs in hypertrophy of ligamentum flavum (HLF) have not been report.

**Methods:**

The integrated analysis of lncRNAs sequencing, bioinformatics analysis and real-time quantitative PCR were used to identify the key lncRNAs involved in HLF progression. Gain- and loss-function experiments were used to explore the functions of lncRNA X inactive specific transcript (XIST) in HLF. Mechanistically, bioinformatics binding site analysis, RNA pull-down, dual-luciferase reporter assay, and rescue experiments were utilized to investigate the mechanism by which XIST acts as a molecular sponge of miR-302b-3p to regulate VEGFA-mediated autophagy.

**Results:**

We identified that XIST was outstandingly upregulated in HLF tissues and cells. Moreover, the up-regulation of XIST strongly correlated with the thinness and fibrosis degree of LF in LSCS patients. Functionally, knockdown of XIST drastically inhibited proliferation, anti-apoptosis, fibrosis and autophagy of HLF cells in vitro and suppressed hypertrophy and fibrosis of LF tissues in vivo. Intestinally, we uncovered that overexpression of XIST significantly promoted proliferation, anti-apoptosis and fibrosis ability of HLF cells by activating autophagy. Mechanistic studies illustrated that XIST directly medullated the VEGFA-mediated autophagy through sponging miR-302b-3p, thereby enhancing the development and progression of HLF.

**Conclusion:**

Our findings highlighted that the XIST/miR-302b-3p/VEGFA-mediated autophagy axis is involved in development and progression of HLF. At the same time, this study will complement the blank of lncRNA expression profiles in HLF, which laid the foundation for further exploration of the relationship between lncRNAs and HLF in the future.

**Supplementary Information:**

The online version contains supplementary material available at 10.1186/s13062-023-00383-9.

## Background

Lumbar spinal canal stenosis (LSCS) is a common degenerative spinal disease in elderly patients, with the typical clinical symptoms of low back pain, radiation-induced lower limb pain, and intermittent claudication [17, 40]. Clinically, LSCS is mostly caused by the degeneration of the lumbar spine, including hypertrophy and relaxation of the ligamentum flavum, degeneration and hypertrophy of the facet joints and the posterior edge of the vertebral body, herniation and prolapse of the intervertebral disc, and other pathological anatomical changes [13, 44]. Among them, the hypertrophy of the ligamentum flavum (HLF) have been determined to contribute to the key etiology of LSCS [44]. Due to the lack of clear pathogenesis, surgical decompression is the only feasible and effective treatment for HLF-induced LSCS, but many LSCS patients with underlying diseases are intolerant to surgery, which makes it impossible for them to undergo surgical decompression to improve the disease [43, 49, 50]. Therefore, it is an urgent need to elucidate the pathogenesis of HLF in order to find effective molecular targets for the treatment of HLF.

The rapid development of transcriptomics and bioinformatics had revealed that non-coding RNAs (ncRNAs) participant in a dramatically variety of biological functions [3]. Importantly, accumulating evidence in recent years has demonstrated that microRNAs (miRNAs) and circular RNAs (circRNAs) play an important role in development of HLF. For example, Li et al. disclosed that miR-10396b-3p inhibited mechanical stress-induced HLF by inhibiting IL-11, suggesting that mechanical stress/miR-10396b-3p/IL-11 axis played a crucial function in HLF [20]. Additionally, recent studies uncovered that miR-4036 levels were related with the ratio of LF/spinal canal area, and upregulation of miR-4306 significantly inhibited the proliferation and fibrosis of HLF cells [5, 49, 50]. Interestingly, Chen et al. identified that dysregulated circRNAs in HLF were mainly enriched in autophagy and mTOR signaling pathways by high-throughput sequencing, and demonstrated that overexpression of hsa_circ_0052318 inhibited fibrosis of LF cells from HLF tissue [4]. Therefore, the in-depth analysis of ncRNAs will help to further fully elucidate the pathogenesis of HLF at the epigenetic level.

Long non-coding RNAs (lncRNAs) were a kind of ncRNA with more than 200 nucleotides and lack of ability to encode proteins [1]. Current studies have shown that lncRNAs can regulate a great diversity of biological functions at the epigenetic, transcriptional and post-transcriptional levels, protein/RNA stability and translation, thereby affecting the occurrence and development of multiple disorders [9, 15, 19, 21, 32]. However, few lncRNAs associated with HLF have been reported so far. In the current study, we identified that lncRNA X inactive specific transcript (XIST) was strongly linked to the HLF development in patients with LSCS through integrated analysis of high-throughput sequencing technology, bioinformatics analysis and real-time quantitative PCR (RT-qPCR). Further, we evaluated the biological function and underlying mechanisms of XIST in HLF development from multiple perspectives. Our findings would elucidate the novel pathogenesis of HLF and provide promising therapeutic targets for HLF-induced LSCS.

## Materials and methods

### Collection of human LF samples

The LF tissues were collected from patients with LSCS or lumbar disc herniation (LDH) under-going lumbar spine surgery in Zhujiang Hospital of Southern Medical University. Some of LF tissues obtained during surgery were quickly frozen in liquid nitrogen and then stored at – 80 °C for later sequencing and molecular biological analysis, while the remaining tissues were immediately used for isolation of primary LF cells. The inclusion criteria of HLF tissue samples were LF thickness greater than 4 mm in the L4/5 level of LSCS patients on magnetic resonance imaging (MRI) scan, and the inclusion criteria of control tissue samples were LF thickness of less than or equal to 4 mm in the L4/5 level of LDH patient on MRI scan. In addition, cancer, heart disease, kidney disease, rheumatic disease and autoimmune disease were excluded from all enrolled patients. The information of all enrolled patients in this study had been summarized in Additional file 1: Table S1. This study was approved by the Institutional Research Ethics Committee of the Zhujiang Hospital of Southern Medical University (Approval No.: 2021-ky-122-01), and informed consent was acquired from all enrolled patients before conducting the study.

### RNA sequencing and analysis

RNA sequencing and original data analysis were commissioned by SHANGHAI BIOTECHNOLOGY CORPORATION (Shanghai, China). For lncRNAs and mRNAs sequencing, purified total RNAs from HLF and control tissues were used for library construction with VAHTS Total RNA-Seq (H/M/R) Library Prep Kit (Illumina, Inc.), and then sequenced using an Illumina HiSeq Xten platform. Clean reads filtered from the raw reads using Seqtk were aligned to the reference genome (Homo sapiens. GRCh38) by spliced mapping algorithm of Hisat2 (Version: 2.0.4). For miRNA sequencing, purified total RNAs from HLF and control tissues were used for library construction with NEBNext^®^ Small RNA Library Prep Kits (Illumina, Inc.), and then sequenced with an Illumina HiSeq Xten platform. Clean reads (18–40 nt) filtered from the raw reads using fastx (Version: 0.0.13) were aligned to the reference genome (Homo sapiens, miRBase) by spliced mapping algorithm of Bowtie, while the miRCat tool in the sRNA Toolkit package was used to predict novel miRNAs. Differentially expressed RNAs between HLF and non-HLF tissues were analyzed using edgeR software, and the screening criteria were |log2 (fold change)|> 1 and p-value < 0.05. Volcano plots were used to identify differentially expressed RNAs.

### Bioinformatics analysis

The data analysis flowchart was shown in Additional file 1: Fig. S1A. Firstly, the differentially expressed miRNAs-targeted lncRNAs were predicted using the lncBaseV2 database, and then the intersections were taken with the differentially expressed lncRNAs. Secondly, the differentially expressed miRNAs-targeted mRNAs were predicted using both starbase database, and then the intersections were taken with the differentially expressed mRNAs. Thirdly, we used Cytoscape to constructed the lncRNAs-miRNAs-mRNAs ceRNA network according to the principle of ceRNA regulatory mechanism. Fourthly, STRING online website was used to analyze protein–protein interaction (PPI) among differentially expressed mRNAs in the ceRNA network, and then the hub genes of the PPI network were identified by CytoHubba software after removing the points with no interaction. Finally, we constructed the hub mRNAs-miRNAs-lncRNAs ceRNA network using Cytoscape.

### RT-qPCR

Total RNA was extracted using Trizol reagents (Invitrogen, Carlsbad, CA), followed by reverse transcription and RT-qPCR amplification as described in our previous studies [2, 5]. U6 and GAPDH were used as an internal control for miRNAs and other RNA, respectively. The RNA expression level was evaluated by 2^−ΔΔCT^ method. The sequence of all primers in this study was listed in Additional file 1: Table S2.

### Isolation and identification of primary LF cells

In terms of our previously described method [2, 5], LF cells were isolated from LF tissues of LSCS or LDH patients. Briefly, the collected LF samples were cut into small pieces and digested for 2 h at 37 °C by Dulbecco’s modified Eagle’s medium (DMEM, Gibco) with 0.2% type I collagenase (Gibco). After digested, the cells were isolated by the DMEM containing 10% fetal bovine serum (FBS, Gibco) and 1% penicillin/streptomycin (Invitrogen). The obtained cells with third passages were identified as LF cells by observing cell morphology under light microscope and evaluating the positive rates of specific markers [collagen I (1:100, #72026, Cell Signal Technology) and fibronectin (1:50, ab237287, Abcam)] using immunofluorescence. Finally, the LF cells from the third to fifth passages were used for subsequent studies.

### Adenovirus construction and infection

The adenoviruses overexpressing and interfering with XIST or VEGFA and their negative control were obtained from Genechem (Nanjing, China). The overexpressing and interfering with miR-302b-3p and their negative control were derived from Ribobio Inc. (Guangzhou, China). Additionally, miR-302b-3p mimics, miR-302b-3p inhibitor and their negative control were obtained from Genechem (Nanjing, China). The cells were infected or transfected with the indicated vectors and shRNAs according the manufacturer’s instructions, and the infection or transfection efficiency was verified by RT-qPCR. The sequences of all shRNAs and miR-302b-3p mimics/inhibitor were listed in Additional file 1: Table S3.

### CCK-8 assay

CCK-8 assay was used to evaluate proliferation ability of cells. Briefly, the treated cells were dealt with enhanced Cell Counting Kit-8 (Beyotime, Shanghai, China) according to operating instruction, and then the optical density (OD) values were determined at 450 nm using a microplate reader (Tecan, F50).

### Flow cytometry

Annexin V-FITC Apoptosis Detection Kit (Beyotime, Shanghai, China) was utilized to evaluate cell apoptosis according to operation instructions. In brief, the treated cells were mixed with Annexin V-FITC staining solution & propidium iodide staining solution, and then incubated in the dark at room temperature. After 20 min of incubation, cell apoptosis was detected by flow cytometry (FACSVantage SE, BD, USA) immediately.

### Western blot

Western blot was performed as described in our previous studies [2]. In brief, total protein was extracted using pre-cooled RIPA lysis buffer (Beyotime, Shanghai, China) containing PMSF and phosphatase inhibitor, and then total protein concentration was measured by a BCA protein quantification kit (Beyotime, Shanghai, China). After denaturation, equal amounts of total protein were separated on sodium dodecyl sulfate polyacrylamide gel electrophoresis (SDS-PAGE) gels and then transferred to polyvinylidene fluoride (PVDF) membranes (Millipore, USA). Subsequently, the PVDF membrane was sealed with 5% skim milk for 1 h, and then incubated with primary antibodies for overnight at 4 °C. After overnight incubation of the primary antibody, the PDVF membrane was incubated with the secondary antibody for 2 h at room temperature. Finally, immunoreactive protein bands were visualized and analyzed by the ECL kit (Pierce, Thermo Fisher Scientific, IL, USA) and ImageJ. Primary antibody information was as follows: cleaved caspase-3 (1:500, ab32042, Abcam), Bax (1:1000, ab182733, Abcam), Bcl-2 (1:500, ab182858, Abcam), VEGFA (1:1000, ab214424), collagen I (1:1000, ab260043, Abcam), collagen III (1:1000, ab7778, Abcam), MMP2 (1:500, ab181286, Abcam), MMP13 (1:1000, ab39012, Abcam), GAPDH (1:1000, ab8245, Abcam), and β-actin (1:2000, ab8227, Abcam).

### RNA fluorescence in situ hybridization (RNA FISH)

RNA FISH was used to analyze the subcellular localization of XIST in LF cells. In brief, fixed and permeable LF cells were treated using RNA FISH SA-Biotin Amplification System Kit (GenePharma, Shanghai, China) according to operating instructions. Subsequently, the nuclei were repopulated using a 4,6-diamidino-2-phenylindole (DAPI) staining solution. Finally, inverted fluorescence microscopy (Mshot, MF52) was used to observe and collect fluorescence images.

### Nuclear and cytoplasmic RNA fractionation analysis

Nuclear and cytoplasmic RNA were extracted from LF cells using Cytoplasmic & Nuclear RNA Purification Kit (Ambion, AM1921) according to the manufacturer's protocol, and then gene expression was analyzed by RT-qPCR. GAPDH and U6 acted as cytoplasmic and nuclear controls, respectively.

### RNA pull-down assay

RNA pull-down assay was performed using the RNA Pull-Down Kit (Thermo Scientific, USA) according to the operating instructions. In short, LF cells were lysed with using the precooled capture buffer containing RNase inhibitors and protease inhibitors, and then incubated with streptavidin-coated magnetic beads/biotin-labeled 302b-3p probe (GenePharma, Shanghai, China) or control probe (GenePharma, Shanghai, China) for 1 h at 4 °C to pull down biotin-labeled miR-302b-3p complexes. Subsequently, the complexes were washed three times with a wash buffer containing RNase inhibitors and protease inhibitors, and then total RNA in the complexes was extracted using TRIzol (Takara, Dalian, China). Finally, abundance levels of XIST or VEGFA were measured by RT-qPCR.

### Dual-luciferase reporter assay

Dual-luciferase reporter assay was used to determine the specific binding sites between miR-302b-3P and XIST or VEGFA 3’UTR. The sequences of XIST and VEGFA-3’UTR and their corresponding mutant versions without miR-302b-3p binding sites were synthesized and then subcloned into luciferase reporter vector, named the XIST-WT, XIST-Mut, VEGFA 3’UTR-WT and VEGFA 3’UTR-Mut, respectively. These luciferase reporter vectors were then co-transfected with miR-302b-3p mimics or negative control (NC) mimics into 293 T cells, respectively. Finally, relative luciferase activity was assessed by a Dual Luciferase Assay Kit (Promega, Madison, WI, USA) in accordance with operating instructions. Renilla luciferase activity served as quality control.

### Animal experiments

All animal experimental protocols were approved by the Ethics Committee for Animal Research of Zhujiang Hospital of Southern Medical University (Guangzhou, China). All C57BL/6 mice (8-week-old, male, SPF) were purchased from the Experimental Animal Center of Southern Medical University (Guangzhou, China). The HLF mouse model was constructed according to the hydrophobic characteristics of previously described mice [5]. All mice were randomly assigned to the control group (n = 5), HLF model group (n = 5), HLF + AAV-shNC group (n = 5), and HLF + AAV-shXIST group (n = 5). After the seventh week of modeling, the skin of the mice lumbar vertebrae was longitudinally dissected, and the dorsal paravertebral muscles were removed from the spinous process and lamina to fully expose the ligamentum flavum at L5/6. Subsequently, AAV-shNC or AAV-shXIST (1 × 10^12^ vg/ml, 3 µl) from Hambo Biotechnology (Shanghai, China) was injected into the ligamum flavum at L5/6 of anesthetized mice using a microinjector (NF36BV 36GA, NanoFil, United States) under an operating microscope. After 4 weeks of AVV injection, the mice were euthanized and the L5/6 vertebrae were collected to obtain LF tissue for later histological analysis and molecular biological analysis.

### Histological analyses

Hematoxylin and eosin (H&E) staining and Elastica van Gieson (EVG) staining were used to evaluate the LF areas and LF fibrosis degree, respectively. Paraffin sections, HE staining, and EVG staining of LF tissue were performed according to our previously reported methods [5]. ImageJ software (NIH, USA) was used to quantify LF areas and LF fibrosis degree (the ratio of elastic fibers to collagen fibers).

### Statistical analysis

All statistical analyses were performed using GraphPad Prism 6.0 (GraphPad Software, La Jolla, USA). Statistical analyses between groups were performed using Student’s t-test and a one-way ANOVA with Tukey's multiple comparison test. The Spearman’s correlation coefficient was used to analyze the correlation between the two indices. All data were performed at least three independent experiments and shown as means ± standard deviation (SD). The p-value < 0.05 was determined to be a significant difference.

## Results

### XIST was upregulated in HLF tissue and associated with LF thinness and fibrosis

**Fig. 1:**
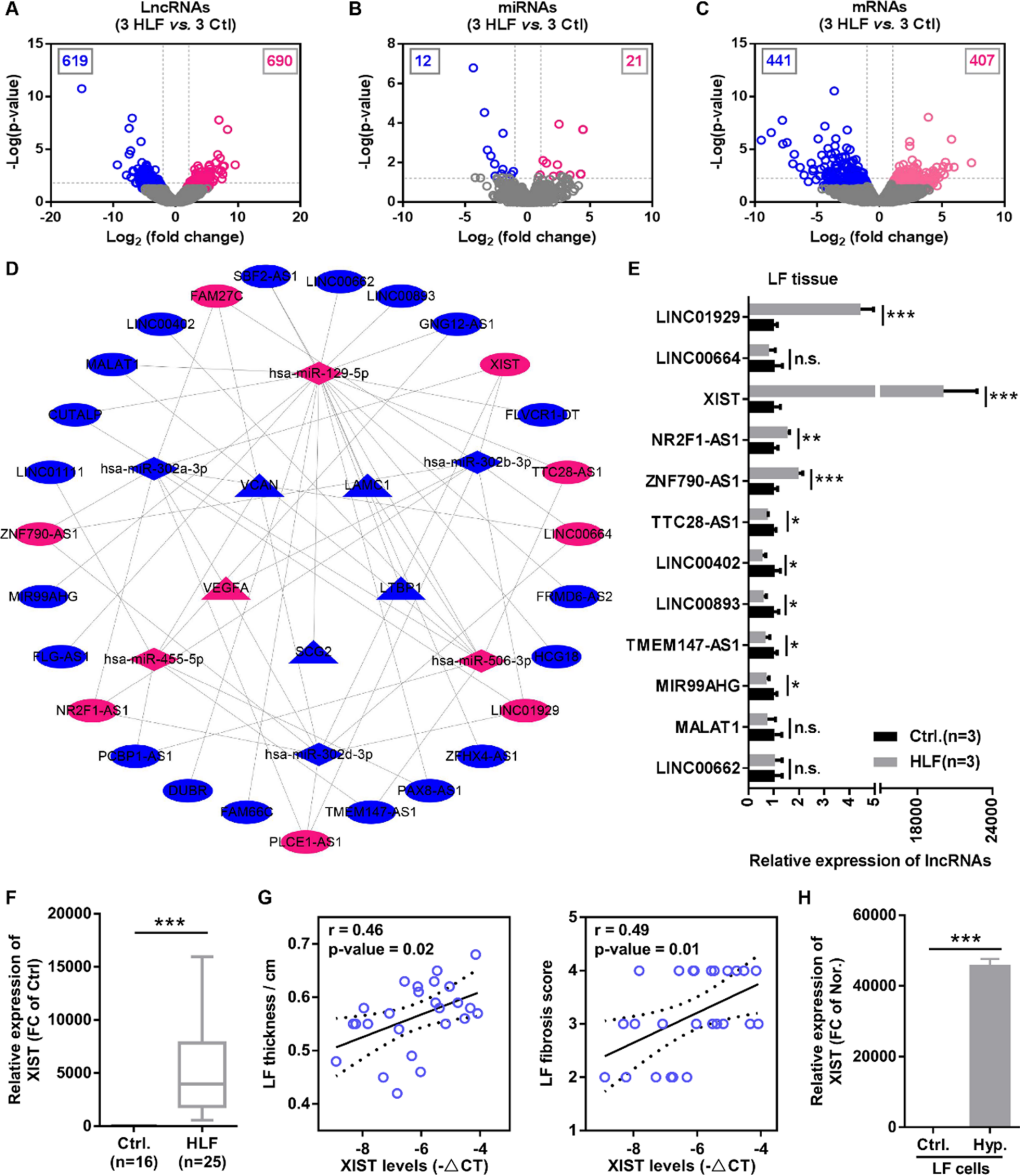
XIST upregulated in HLF tissues and associated with the LF hypertrophy and fibrosis. **A**–**C** The volcano plots visualized the expression of lncRNAs, miRNAs and mRNAs between 3 HLF tissues and 3 control tissues. The red and blue dots in the volcano plots indicated statistically significant upregulated and downregulated RNAs, respectively. **D** Construction of hub mRNAs-miRNAs-lncRNAs competing endogenous RNA (ceRNA) networks in HLF. Ellipses, quadrangle, and triangles represented dysregulated lncRNAs, dysregulated miRNAs, and dysregulated hub mRNAs, respectively. Pink and blue represented upregulated and downregulated RNAs, respectively. **E** RT-qPCR was used to verify the expression of dysregulated lncRNAs from the ceRNA network in 3 HLF tissues and 3 control tissues. **F** The XIST expression in 25 HLF tissues and 16 control tissues was detected by RT-qPCR, and found that XIST was upregulated in HLF tissues. **G** Pearson correlation coefficient was used to analyze the correlation between the XIST expression and LF hypertrophy or LF fibrosis.** H** RT-qPCR was used to measure the XIST expression in normal LF tissue-derived cells (non-HLF cells) and HLF tissues-derived cells (HLF cells), and found that XIST expression levels was markedly upregulated in HLF cells compared to non-HLF cells. ***P < 0.001

To investigate the expression profiles of lncRNA, miRNA and mRNA in patients with HLF, RNA-seq was performed in HLF tissues (n = 3) and control tissues (n = 3), and |logFC|> 1.0 and P-value < 0.05 was used as a screening criterion for differentially expressed RNAs. We found that a total of 1309 lncRNAs were differentially expressed between HLF tissues and control tissues, among which 690 were upregulated and 619 were downregulated (Fig. 1A). Compared with control tissues, 33 miRNAs were dysregulated in HLF tissue, including 21 miRNAs upregulated and 11 miRNAs downregulated (Fig. 1B). Moreover, we identified 847 differentially expressed mRNAs between HLF tissue and control tissue, among which 407 mRNAs were upregulated and 441 mRNAs were downregulated in HLF tissue (Fig. 1C). According to the ceRNA hypothesis, we undertook an integrative analysis of the differentially expressed lncRNAs, miRNAs, and mRNAs to construct a lncRNAs -miRNAs - mRNAs ceRNAs network in HLF. We found that 40 lncRNAs, 9 miRNAs, and 90 mRNAs participated in the formation of ceRNA (Addtional file 1: Fig. S1B). The differentially expressed mRNAs in ceRNA network were further analyzed by PPI and cytoHubba, and five hub genes (LAMC1, LTBP1, VCAN, SCG2, and VEGFA) were identified (Addtional file 1: Fig. S1C). Finally, we further constructed hub mRNAs (5 DEmRNAs) - miRNAs (6 DEmiRNAs) - lncRNAs (27 DElncRNAs) ceRNA network in HLF through a series of bioinformatics analyses (Fig. 1D). These data not only described the expression profile of lncRNAs in HLF for the first time, but also constructed the hub mRNAs-miRNAs-lncRNAs ceRNA network in HLF, which provided the necessary support for later studies on the involvement of lncRNAs in HLF pathogenesis.

In addition, we randomly selected 12 lncRNAs from the hub mRNAs-miRNAs-lncRNAs ceRNA network and then examined their expression levels in the RNA-seq samples using RT-qPCR. The results showed that except for LINC00664, MALAT1, and LINC00662, the detection results of other lncRNAs were consistent with the RNA-seq data, among which XIST showed the most significant difference (Fig. 1E). Expanding the clinical samples, we further determined that XIST was markedly upregulated in LSCS patients with HLF (Fig. 1F). Pearson correlation coefficient analysis showed that XIST expression levels were positively correlated with LF thickness and fibrosis in LSCS patient with HLF (Fig. 1G). Furthermore, the cells isolated from the LF tissue were identified by microscopic observation and immunofluorescence, and found that the third passage of cells had typical morphology of LF cells and uniformly expressed LF cells markers (collagen I and fibronectin), implying that these cells were all high purity of the LF cells (Additional file 1: Fig. S2). Subsequently, the XIST expression levels were examined between control LF tissue-derived cells (named non-HLF cells) and HLF tissues-derived cells (named HLF cells) by RT-qPCR. In accordance with our measurements in tissues, the results indicated that XIST expression level was markedly upregulated in HLF cells compared with non-HLF cells (Fig. 1H). Taken together, our results suggested that the XIST-mediated ceRNA network might play a critical role in HLF pathogenesis.

### XIST aggravated HLF cells proliferation, anti-apoptosis, and fibrosis in vitro

**Fig. 2:**
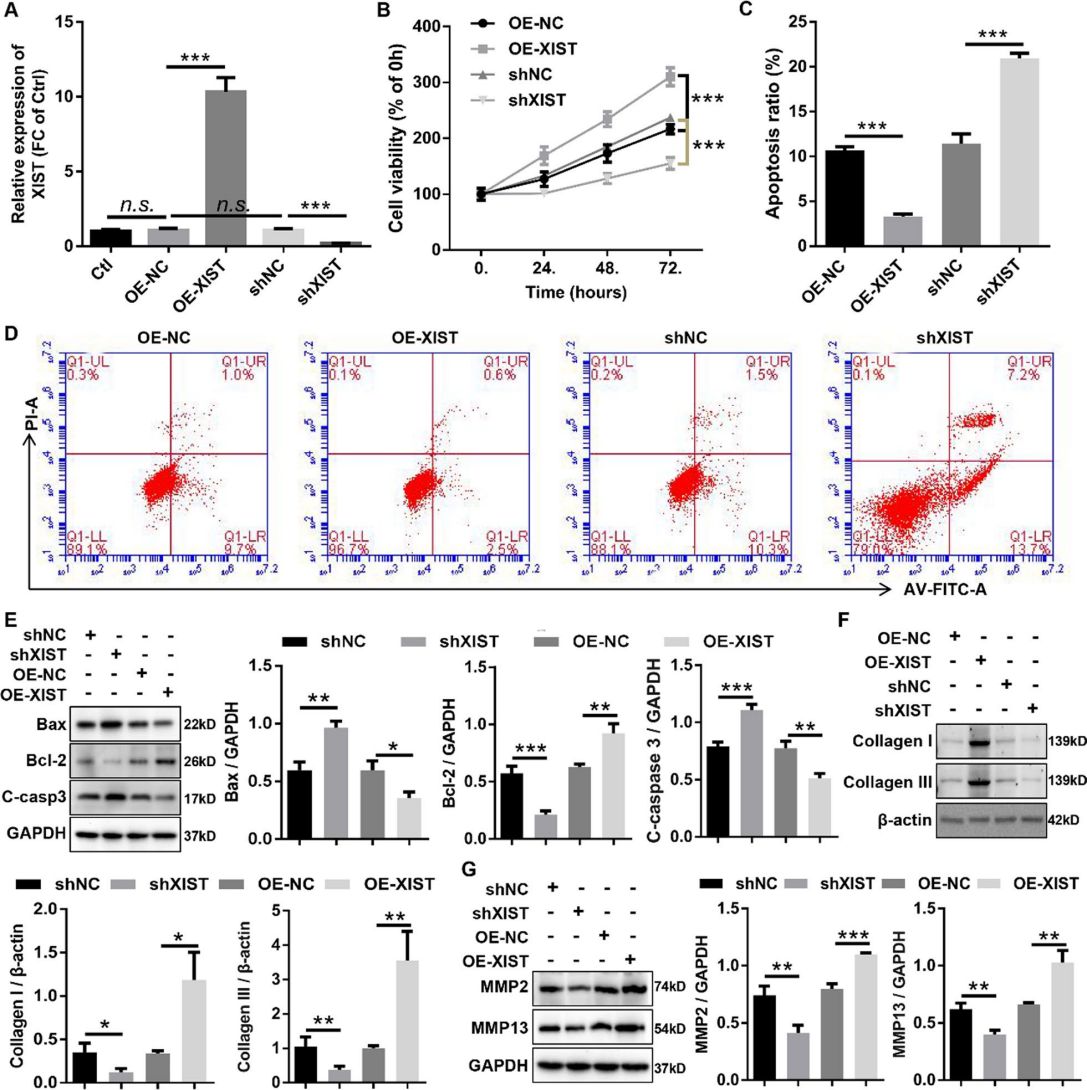
XIST aggravated HLF cells proliferation, anti-apoptosis, and fibrosis in vitro. **A** HLF cells were infected with XIST overexpression adenovirus and RNAi adenovirus against XIST, and the expression of XIST was confirmed by RT-qPCR. **B** Growth curves of HLF cells infected with the indicated adenovirus was evaluated by CCK-8 assays. **C**/**D** The apoptosis ratios of HLF cells infected with the indicated adenovirus were assessed by flow cytometry with Annexin V/PI double staining. **E** The protein expression of apoptosis-related genes (cleaved caspase3, Bax, and Bcl-2) in HLF cells infected with the indicated adenovirus was measured by western blotting. **F** and **G** The protein expression of fibrosis-related genes (collagen I, collagen III, MMP2, and MMP13) in HLF cells infected with the indicated adenovirus was confirmed by western blotting. C-caspase 3, cleaved-caspase3; shNC, corresponding negative control of shXIST; shXIST, the RNAi adenovirus against XIST; OE-NC, corresponding negative control of OE-XIST; OE-XIST, the overexpression adenovirus of XIST. ^*^P < 0.05, ^**^P < 0.01, and ^***^P < 0.001

To explore the biological function of XIST in HLF cells, overexpression adenovirus of XIST (OE-XIST) and RNAi adenovirus against XIST (shXIST) were constructed. The results showed that XIST was overexpressed and silenced in HLF cells infected with OE-XIST and shXIST by RT-qPCR (Fig. 2A). Growth curves executed by CCK8 assays uncovered that overexpression of XIST significantly promoted the proliferation viability of HLF cells, whereas knockdown of XIST inhibited the proliferation viability of HLF cells (Fig. 2B). Apoptosis detection by flow cytometry analysis with Annexin V/PI double staining further demonstrated that the apoptosis ratios of HLF cells were significantly decreased by upregulation of XIST, while downregulation of XIST displayed an opposite effect (Fig. 2C, D). Meanwhile, western blotting was carried out to evaluate the effect of XIST on the expression levels of apoptosis-related proteins in HLF cells. The results demonstrated that HLF cells transfected with OE-XIST displayed lower expression of proapoptotic proteins (Bax and cleaved caspase-3) as well as higher expression of antiapoptotic protein (Bcl-2) than HLF cells transfected with OE-NC (Fig. 2E). On the contrary, inhibition of XIST in HLF cells resulted in a significantly enhancement in the expression of proapoptotic protein (Bax and cleaved caspase-3) and an obviously decreased in the expression of antiapoptotic protein (Bcl-2) in HLF cells (Fig. 2E). This data suggested that upregulation of XIST suppressed apoptosis in HLF cells, while downregulation of XIST promoted apoptosis in HLF cells. In addition, fibrosis has been proved to be the crucial pathological characterization of HLF [34]. To investigate whether XIST has an effect on fibrosis of HLF cells, western blotting was carried out to detect expression of fibrosis-related proteins (collagen I, collagen III, MMP2, and MMP13). The results revealed that overexpression of XIST outstandingly enhanced protein expression of collagen I, collagen I, MMP2, and MMP13 in HLF cells, whereas knockdown of XIST dramatically suppressed protein expression of collagen I, collagen I, MMP2, and MMP13 (Fig. 2F, G). This experiment suggested that the fibrosis of HLF cells was markedly increased by overexpression of XIST, and dramatically decreased by knockdown of XIST. Taken together, our data suggested that XIST aggravated the pathological progression of HLF cells in vitro, whereas knockdown of XIST inhibited the pathological progression of HLF cells in vitro.

### Knockdown of XIST inhibited LF hyperplastic fibrosis in vivo

**Fig. 3:**
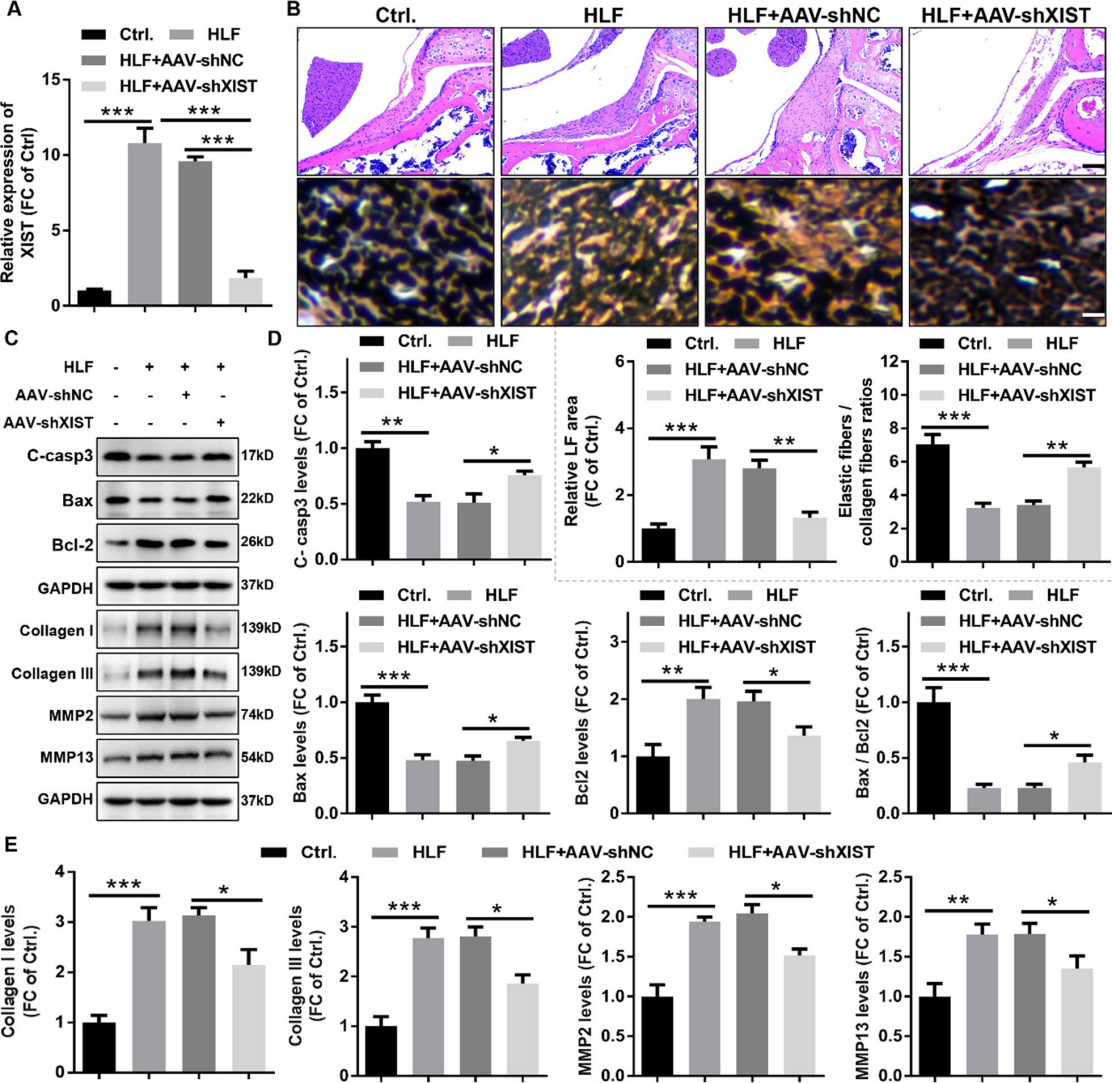
XIST knockdown inhibited LF hyperplastic fibrosis in vivo. **A** XIST expression in LF tissue from different treated mice was determined by RT-qPCR. **B** Representative images of H&E staining of the LF and quantitative analysis of the LF area. Scale bar = 100 μm. Representative images of EVG staining of the LF and quantitative analysis of elastic fiber area to collagen fiber area. Scale bar = 20 μm. **C** and **D** The expression of apoptosis-related proteins (cleaved caspase3, Bax, and Bcl-2) in LF tissues from different treated mice were measured by western blotting. **C** and **E** The expression of fibrosis-related proteins (collagen I, collagen III, MMP2, and MMP13) in LF tissues from different treated mice were evaluated by western blotting. Ctrl, control mice; HLF, HLF mice induced by bipedal standing; AAV-shNC, corresponding negative control of AAV-shTCF7; AAV-shTCF7, the RNAi adeno associated virus against XIST. ^*^P < 0.05, ^**^P < 0.01, and.^***^P < 0.001

To confirm the effects of XIST on LF hyperplastic fibrosis in vivo, the RNAi adeno-associated virus against XIST (AAV-shXIST) and its negative control (AAV-NC) were constructed and then injected into the LF at L5/6 of anesthetized mice with HLF using a microinjector. The results of RT-qPCR uncovered that XIST was significantly enhanced in LF tissue of HLF mice compared with LF tissue of control mice, while the XIST expression was outstandingly decreased in HLF mice treated with AAV-shXIST compared with HLF mice treated with or without AVV-shNC (Fig. 3A). Meanwhile, we found that AVV-NC treatment had no effect on XIST expression in LF tissue of mice with HLF (Fig. 3A). HE staining and EVG staining displayed that LF areas and fibrosis degree were obviously enhanced in HLF mice, and this effect was inhibited by AVV-shXIST treatment (Fig. 3B). In addition, western blotting was used to evaluate the effect of XIST on apoptosis-related protein in LF tissue of mice with HLF. The results indicated that LF tissue of mice with HLF had a higher expression of proapoptotic proteins (Bax and cleaved caspase-3) as well as a lower expression of antiapoptotic protein (Bcl-2) than LF tissue of control mice, and this effect was obviously suppressed by AAV-shXIST treatment (Fig. 3C, D). Finally, western blotting was used to confirm the effect of XIST on fibrosis progression of HLF. The results showed that the protein expression levels of collagen I, collagen I, MMP2, and MMP13 were dramatically upregulated in LF tissue of mice with HLF, whereas this function was significantly reversed by AAV-shXIST treatment (Fig. 3C, E). In addition, we found no changes in LF area and fibrosis in HLF mice treated with AVV-shNC compared with HLF mice (Fig. 3B, C, D, and E), suggesting that AAV-shNC treatment had no effect on progression of HLF. In short, this data confirmed the pro-fibrotic role of XIST in HLF, suggesting that targeted inhibition of XIST expression might be a potential therapeutic strategy for LSCS patients with HLF.

### XIST promoted the pathological progression of HLF cells by activating autophagy

**Fig. 4:**
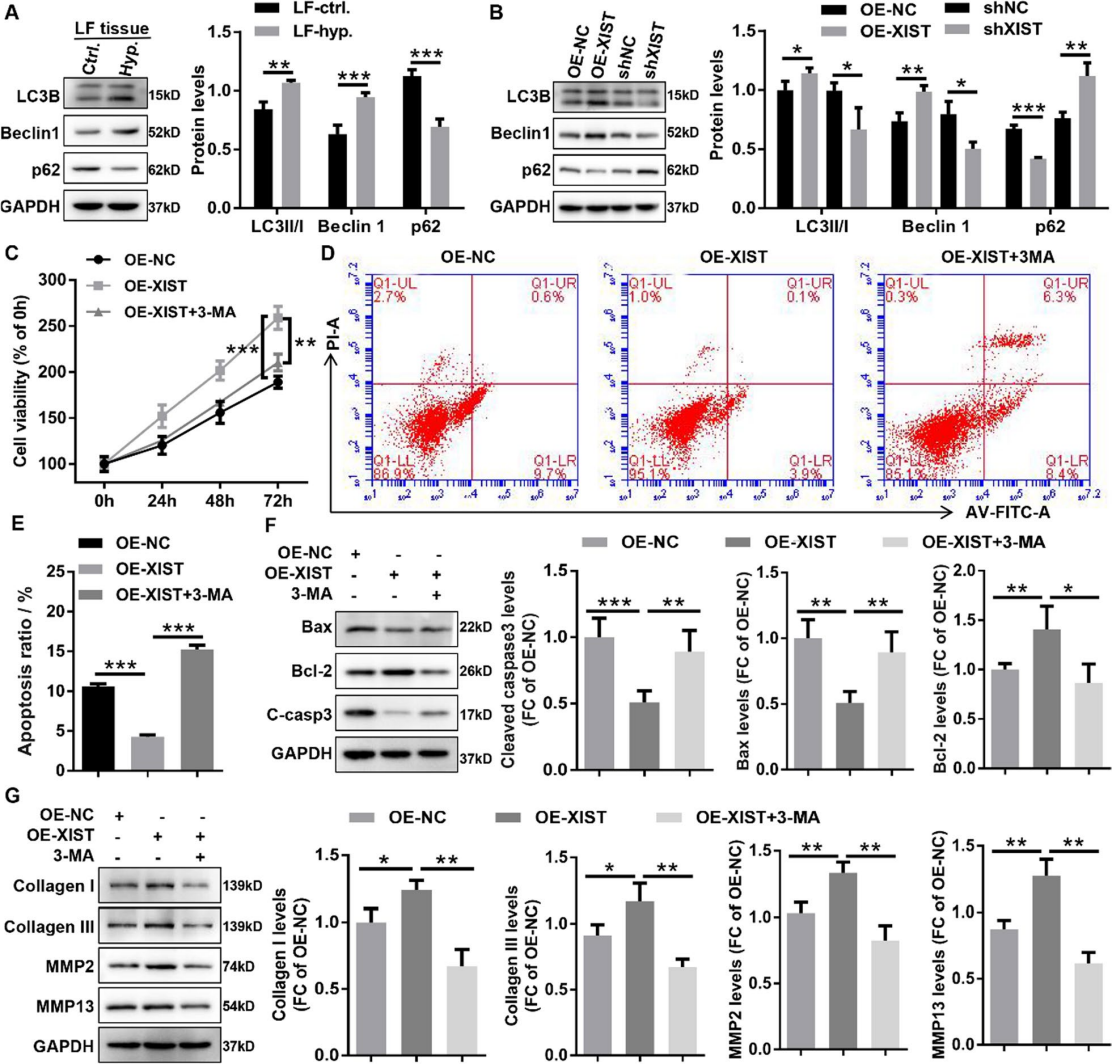
XIST promoted the pathological progression of HLF cells by activating autophagy. **A** The expression levels of autophagy-related proteins (LC3B, Beclin1, and p62) in LF tissues from patients with or without HLF were evaluated by western blotting. **B** The expression levels of autophagy-related protein (LC3B, Beclin1, and p62) in HLF cells infected with the indicated adenovirus were evaluated by western blotting. **C** The growth curves of HLF cells treated with the indicated adenovirus or 3-MA was evaluated by CCK-8 assays. **D** and **E** The apoptosis ratios of HLF cells treated with the indicated adenovirus or 3-MA were assessed by flow cytometry with Annexin V/PI double staining. **F** The protein expression of apoptosis-related genes (cleaved caspase3, Bax, and Bcl-2) in HLF cells treated with the indicated adenovirus or 3-MA was measured by western blotting. **G** The protein expression of fibrosis-related genes (collagen I, collagen III, MMP2, and MMP13) in HLF cells treated with indicated adenovirus or 3-MA was confirmed by western blotting. C-casp3, cleaved-caspase-3; shNC, corresponding negative control of shXIST; shXIST, the RNAi adenovirus against XIST; OE-NC, corresponding negative control of OE-XIST; OE-XIST, the overexpression adenovirus of XIST; 3-MA, autophagy inhibitor 3-methyladenine. ^*^P < 0.05, ^**^P < 0.01, and.^***^P < 0.001

Previous studies have shown that autophagy may be involved in HLF progression [4], and is closely related to fibrosis [48, 51]. To further determine whether autophagy was involved in HLF progression, western blotting was used to detect the expression levels of autophagy marker proteins in human HLF tissues and control tissues. The results indicated that human HLF tissue had a higher LC3II/LC3I ratio and expression of Beclin 1 protein as well as a lower expression of p62 protein compared with human normal tissue (Fig. 4A). Compared with the control mice, the LC3II/LC3I ratio and Beclin 1 protein expression were significantly increased, while p62 protein expression was obviously decreased in the HLF mice (Additional file 1: Fig. S3), revealing that autophagy was activated in the LF tissue of HLF mice. In addition, extensive evidence has shown that XIST can activate autophagy and participate in a variety of disease processes [22, 23, 42, 52]. To determine whether XIST is involved in the autophagy progression in HLF, western blotting was performed to detect the effect of XIST on the expression of autophagy-related proteins in HLF cells or tissues. The results revealed that HLF cells infected with OE-XIST resulted in higher LC3II/LC3I and Beclin 1 protein expression as well as lower p62 protein expression compared with HLF cells infected with OE-NC, whereas HLF cells infected with shXIST showed the opposite effect (Fig. 4B). Meanwhile, in vivo experiments also showed that HLF mice treated with AAV-shXIST resulted in the lower LC3II/LC3I ratio and Beclin 1 protein expression as well as the higher p62 protein expression compared with HLF mice treated with AAV-NC (Additional file 1: Fig. S2), suggesting knockdown of XIST could inhibit autophagy activation in mice with HLF. The above results revealed that autophagy was significantly activated in HLF, whereas XIST knockdown inhibited autophagy activation in vivo and in vitro.

Emerging evidence has shown that the autophagy activation in fibroblasts promotes the collagen release, which is a sufficient and necessary condition for inducing tissue fibrosis [48]. Therefore, we hypothesized that XIST might aggravate pathological processes in HLF cells by mediating autophagy activation. To confirm this hypothesis, we conducted a series of rescue experiments using the autophagy inhibitor 3-MA. Growth curves performed by CCK-8 assays revealed that 3-MA treatment in HLF cells dramatically inhibited the proliferation viability induced by XIST overexpression (Fig. 4C). Flow cytometry analysis with Annexin V/PI double staining demonstrated that 3-MA treatment in HLF cells obviously impaired the apoptosis-suppressing effect of XIST overexpression (Fig. 4D, E). Meanwhile, the results of western blotting also uncovered that 3-MA treatment reversed the anti-apoptosis effects induced by XIST overexpression in HLF cells, characterized by the increased expression of proapoptotic protein (Bax and cleaved caspase-3) and the decreased expression of Bcl-2 in the HLF treated with OE-XIST (Fig. 4F). Moreover, we found that 3-MA treatment markedly decreased the protein expression of collagen I, collagen I, MMP2, and MMP13 in the HLF with XIST overexpression by western blotting (Fig. 4G), suggesting that 3-MA treatment rescued the pro-fibrosis effect induced by XIST overexpression in HLF cells. Taken together, these studies revealed that XIST promoted the pathological progression of HLF cells by activating autophagy.

### XIST indirectly regulated VEGFA expression by acting as a ceRNA for miR-302b-3p

**Fig. 5:**
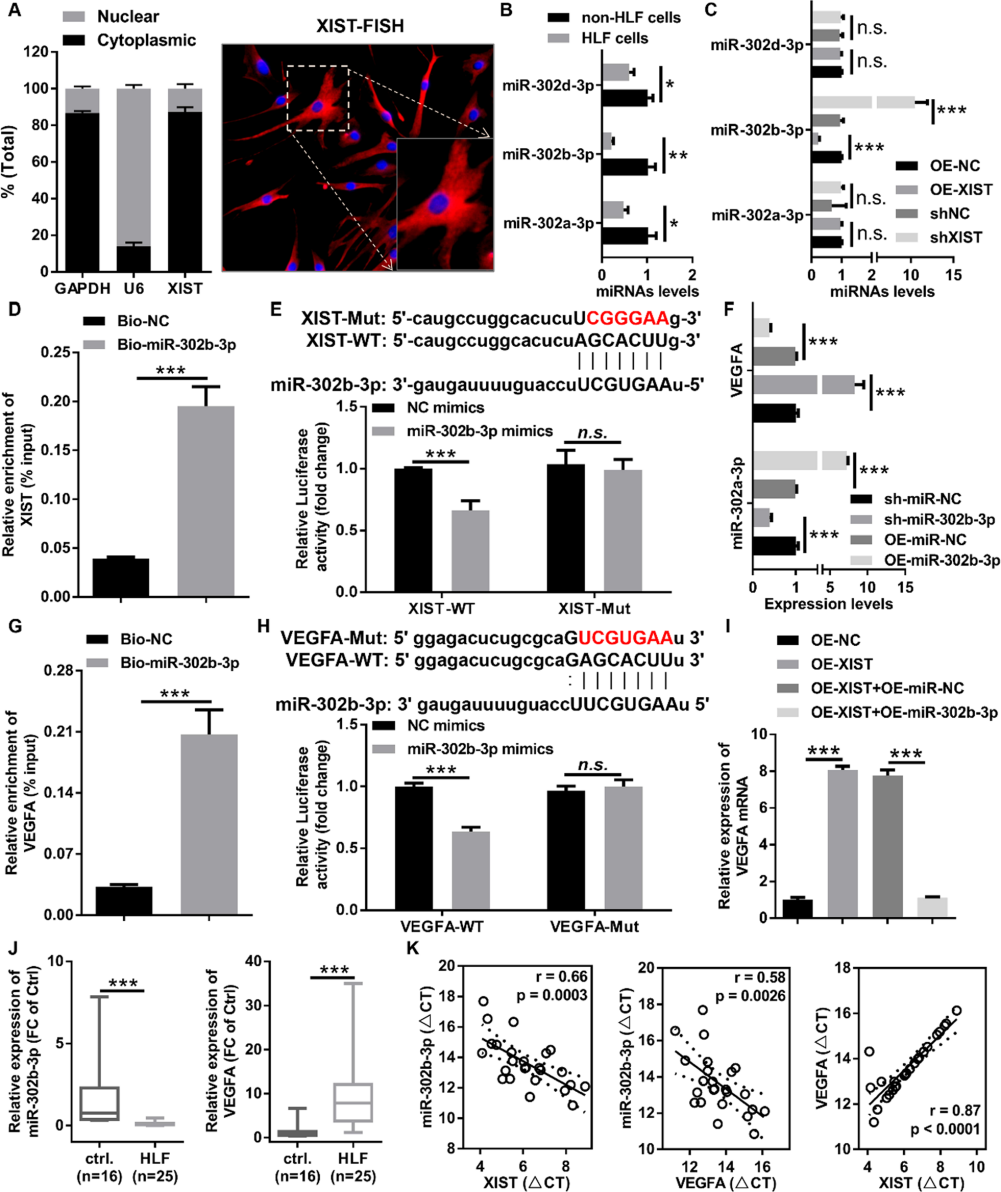
XIST indirectly regulated the VEGFA expression by acting as a ceRNA for miR-302b-3p. **A** Subcellular localization of XIST detected by nuclear and cytoplasmic RNA fractionation analysis and lncRNA FISH. GAPDH and U6 were used as nuclei and cytoplasmic references, respectively. Green fluorescence represented cyc3-labeled XIST probes and blue fluorescence represented DAPI-stained nuclei. **B** The expression of miRNAs (miR-302a-3p, miR-302b-3p, and miR-302d-3p) was evaluated by RT-qPCR in HLF and non-HLF cells. **C** The expression of miRNAs (miR-302a-3p, miR-302b-3p, and miR-302d-3p) was evaluated by RT-qPCR in HLF cells infected with the indicated adenovirus. **D** RNA pull-down was executed in HLF cells, followed by RT-qPCR to detect the enrichment of XIST. **E** The relative luciferase activities were measured in 293 T cells after transfection with XIST-WT or XIST-Mut and miR-302b-3p mimics or NC mimics, respectively. **F** The expression levels of VEGFA and miR-302b-3p were evaluated by RT-qPCR in HLF cells infected with the indicated adenovirus. **G** RNA pull-down was performed in HLF cells, followed by RT-qPCR to detect the enrichment of VEGFA. **H** Relative luciferase activity was measured in 293 T cells after transfection with VEGFA-WT or VEGFA-Mut and miR-302b-3p or NC mimics, respectively. **I** The mRNA expression of VEGFA in HLF cells infected with the indicated adenovirus was evaluated by RT-qPCR. **J** The expression levels of miR-302b-3p and VEGFA in 25 HLF tissues and 16 control LF tissues were detected by RT-qPCR, and VEGFA expression obviously upregulated in HLF tissues and miR-302b-3p expression markedly downregulated in HLF tissues. **K** Pearson correlation coefficient was used to analyze the correlation between the expression levels of XIST, miR-302b-3p, and VEGFA in HLF tissues. shNC, corresponding negative control of shXIST; shXIST, the RNAi adenovirus against XIST; OE-NC, corresponding negative control of OE-XIST; OE-XIST, the overexpression adenovirus of XIST; OE-miR-NC, corresponding negative control of OE-miR-302b-3p; OE-miR-302b-3p, the overexpression adenovirus of miR-302b-3p; sh-miR-NC, corresponding negative control of sh-miR-302b-3p; sh-miR-302b-3p, the RNAi adenovirus against miR-302b-3p. ^*^P < 0.05, ^**^P < 0.01, and.^***^P < 0.001

To elucidate the underlying mechanism of XIST in HLF progression, we determined the localization of XIST in HLF cells by nuclear and cytoplasmic RNA fractionation analysis and lncRNA FISH. The results showed that XIST was mainly localized in the cytoplasm of HLF cells (Fig. 5A), suggesting that XIST might act as the ceRNA mechanism. In the constructed hub mRNA-miRNA-lncRNA ceRNA network, we predicted XIST-miRNAs (miR-302a-3p, miR-302b-3p and miR-302d-3p)-VEGFA axis (Fig. 1D). First, the expression levels of miRNAs (miR-302a-3p, miR-302b-3p, and miR-302d-3p) were examined in non-HLF and HLF cells, and revealed that these miRNAs were significantly downregulated in HLF cells compared with non-HLF cells (Fig. 5B). We then evaluated the effect of XIST on the expression of these miRNAs in HLF cells, and found that XIST negatively regulated miR-302b expression in HLF cells, but had no effect on miR-302a-3p and miR-302d-3p (Fig. 5C). Therefore, we assume that XIST might act as a ceRNA for miR-302b-3p. In order to confirm the predictive bioinformatics analysis, the interaction between XIST and miR-302b was detected using an RNA pull-down assay with a specific biotin-labeled miR-302b-3p probe and a dual-luciferase reporter assay. RNA pull-down assay showed that XIST was specifically enriched in the bio-miR-302b-3p probe group compared with the bio-negative control probe group (Fig. 5D). Dual-luciferase reporter assay indicated that miR-302b-3p mimics markedly reduced the luciferase activity of XIST-WT, but had no effect on the luciferase activity of XIST-Mut (Fig. 5E). Taken all together, this data demonstrated that XIST could act as a sponge for miR-302b-3p in HLF cells.

Additionally, we found that upregulation of miR-302b-3p significantly decreased VEGFA mRNA expression in HLF cells by RT-qPCR, whereas downregulation of miR-302b-3p markedly enhanced VEGFA mRNA expression in HLF cells (Fig. 5F). Subsequently, we conducted RNA pull-down assays with a specific biotin-labeled miR-302b-3p probe and a dual luciferase reporter assay to validate the interaction between VEGFA and miR-302b-3p. RNA pull-down assay showed that a specific enrichment of VEGFA was detected by RT-qPCR in the bio-miR-302b-3p probe group compared with the bio-negative control probe group (Fig. 5G). Dual-luciferase reporter assay indicated that miR-302b-3p mimics markedly decreased the luciferase activity of VEGFA-WT, but had no effect on the luciferase activity of VEGFA-Mut (Fig. 5H). Collectively, this data demonstrated that VEGFA is a target gene for miR-302b-3p in HLF cells.

Finally, to investigate whether XIST upregulated VEGFA expression by mediating miR-302b-3p, rescue experiments were performed using overexpressing or interfering adenovirus of miR-302b-3p. We found that overexpression of XIST markedly enhanced VEGFA expression levels (Fig. 5I), whereas knockdown of XIST strongly decreased VEGFA expression levels (Additional file 1: Fig. S4). As expected, upregulation or downregulation of miR-302b-3p could reverse the enhanced or reduced VEGFA induced by XIST overexpression or knockdown, respectively (Fig. 5I and Additional file 1: Fig. S4). Furthermore, RT-qPCR was used to detect the expression of miR-302b-3p and VEGFA in HLF tissue and normal LF tissue. Compared with normal LF tissue, miR-302b-3p was obviously downregulated and VEGFA was markedly upregulated in HLF tissue (Fig. 5J). Pearson correlation analysis displayed that expression of miR-302b-3p negatively correlated with the expression of XIST and VEGFA in the HLF tissue, and VEGFA expression levels positively correlated with XIST expression levels (Fig. 5K). Collectively, these results demonstrated that XIST regulated VEGFA expression by acting as a ceRNA for miR-302b-3p in HLF cells.

### MiR-302b-3p inhibited HLF cells proliferation, fibrosis and autophagy through mediating VEGFA expression

**Fig. 6:**
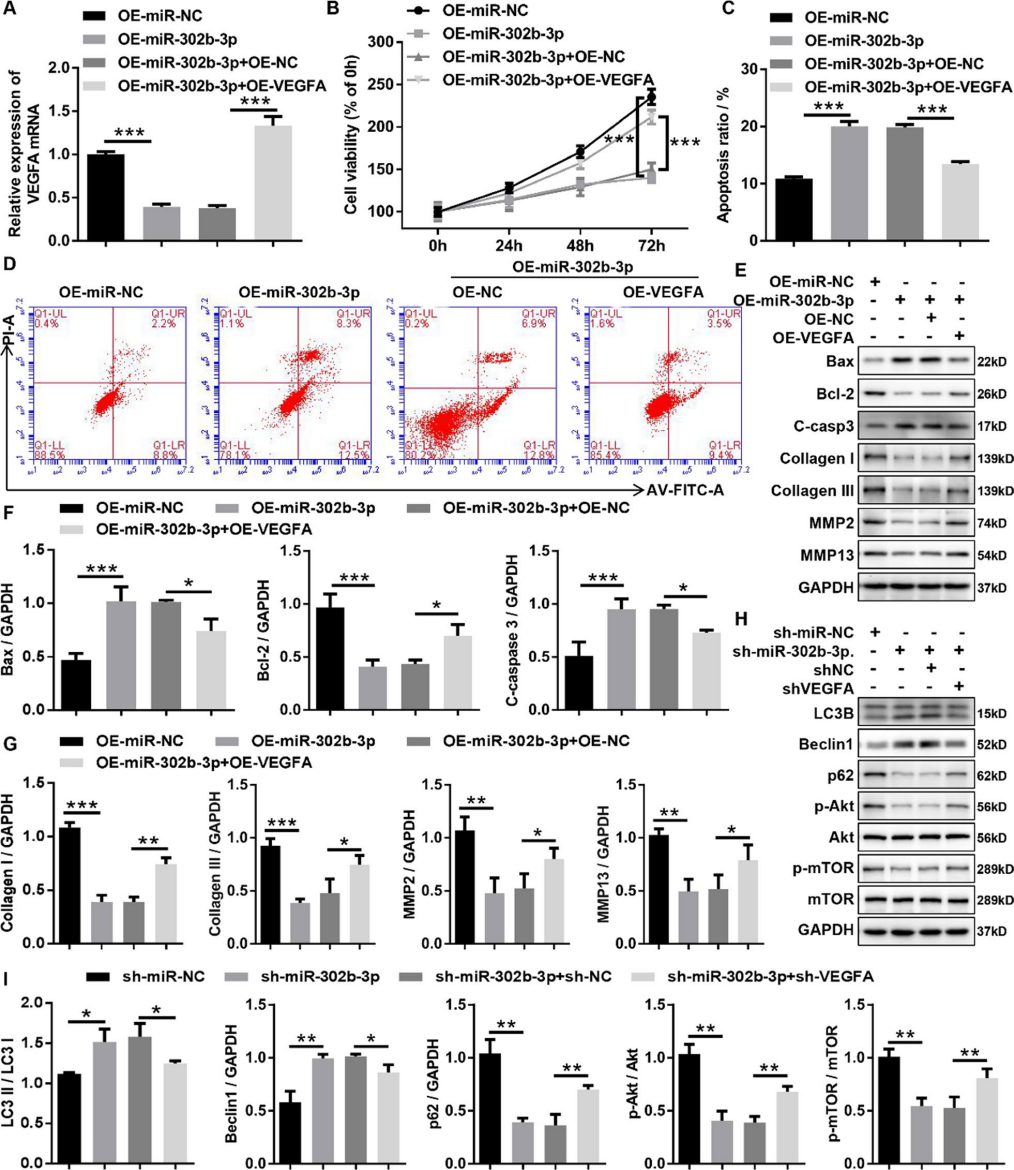
MiR-302b-3p inhibited proliferation, fibrosis, and autophagy through mediating the miR-302b-3p/VEGFA axis in HLF cells. **A** The VEGFA expression in HLF cells infected with the indicated adenovirus was evaluated by RT-qPCR. **B** Growth curves of HLF cells infected with the indicated adenovirus were evaluated by CCK-8 assays. **C** and **D** The apoptosis ratios of HLF cells infected with the indicated adenovirus were assessed by flow cytometry with Annexin V/PI double staining. **E** and **F** The expression of apoptosis-related proteins (cleaved caspase3, Bax, and Bcl-2) in HLF cells infected with indicated adenovirus was measured by western blotting. **E** and **H** The expression of fibrosis-related proteins (collagen I, collagen III, MMP2, and MMP13) in HLF cells infected with the indicated adenovirus was confirmed by western blotting. **G** and **I** The protein expression of autophagy-related proteins (LC3B, Beclin1, p62, p-AKT, Akt, p-mTOR, and mTOR) in HLF cells infected with the indicated adenovirus was confirmed by western blotting. C-casp3, cleaved-caspase-3; shNC, corresponding negative control of shVEGFA; shVEGFA, the RNAi adenovirus against VEGFA; OE-NC, corresponding negative control of OE-VEGFA; OE-VEGFA, the overexpression adenovirus of VEGFA; OE-miR-NC, corresponding negative control of OE-miR-302b-3p; OE-miR-302b-3p, the overexpression adenovirus of miR-302b-3p; sh-miR-NC, corresponding negative control of sh-miR-302b-3p; sh-miR-302b-3p, the RNAi adenovirus against miR-302b-3p. ^*^P < 0.05, ^**^P < 0.01, and.^***^P < 0.001

To explore the biological function of miR-302b-3p/VEGFA axis in HLF cells, HLF cells were infected with miR-302b-3p overexpressing adenovirus (OE- miR-302b-3p) alone or co-infected with VEGFA overexpressing adenovirus (OE-VEGFA). RT-qPCR results displayed that VEGFA overexpression could rescue the inhibition of miR-302b-3p on VEGFA (Fig. 6A). Growth curves performed by CCK-8 assays revealed that miR-302b-3p overexpression significantly inhibited the proliferation viability of HLF cells, whereas upregulation of VEGFA dramatically reduced the proliferation-suppressing effect induced by miR-302b-3p overexpression in HLF cells (Fig. 6B). Flow cytometry analysis with Annexin V/PI double staining demonstrated that miR-302b-3p overexpression significantly promoted the apoptosis capacity of HLF cells, whereas upregulation of VEGFA dramatically impaired the apoptosis-promoting effect induced by overexpression of miR-302b-3p in HLF cells (Fig. 6C, D). Meanwhile, the results of western blotting also uncovered that miR-302b-3p overexpression significantly increased proapoptotic protein (Bax and cleaved caspase-3) expression and decreased Bcl-2 expression in HLF cells, and this effect was reversed by VEGFA overexpression (Fig. 6E, F). In addition, we revealed that miR-302b-3p overexpression significantly decreased the protein expression of collagen I, collagen I, MMP2, and MMP13 in the HLF cells, and this effect was obviously impaired by VEGFA overexpression (Fig. 6E, G). In addition, to investigate whether the miR-302b-3p/VEGFA axis is involved in autophagy in HLF cells, western blotting was used to detect marker proteins and associated signaling pathways for autophagy. Results disclosed that miR-302b-3p knockdown in HLF cells resulted in higher LC3II/LC3I and Beclin 1 protein expression as well as lower p62 protein expression compared with sh-miR-NC, and this effect was obviously reversed by VEGFA knockdown (Fig. 6H, I). Meanwhile, we found that downregulated VEGFA in HLF cells weakened the inhibition effects of miR-302b-3p knockdown on p-Akt/Akt ratio and p-mTOR/mTOR ratio in HLF cells (Fig. 6H, I), suggesting that miR-302b-3p knockdown inhibited Akt/mTOR signaling pathway activation in HLF cells by upregulating VEGFA. Taken together, these studies revealed that miR-302b-3p inhibited proliferation, anti-apoptosis, fibrosis, and autophagy of HLF cells by downregulating VEGFA expression.

### XIST promoted HLF cell proliferation, fibrosis and autophagy through regulating the XIST/miR-302b-3p/VEGFA axis

**Fig. 7:**
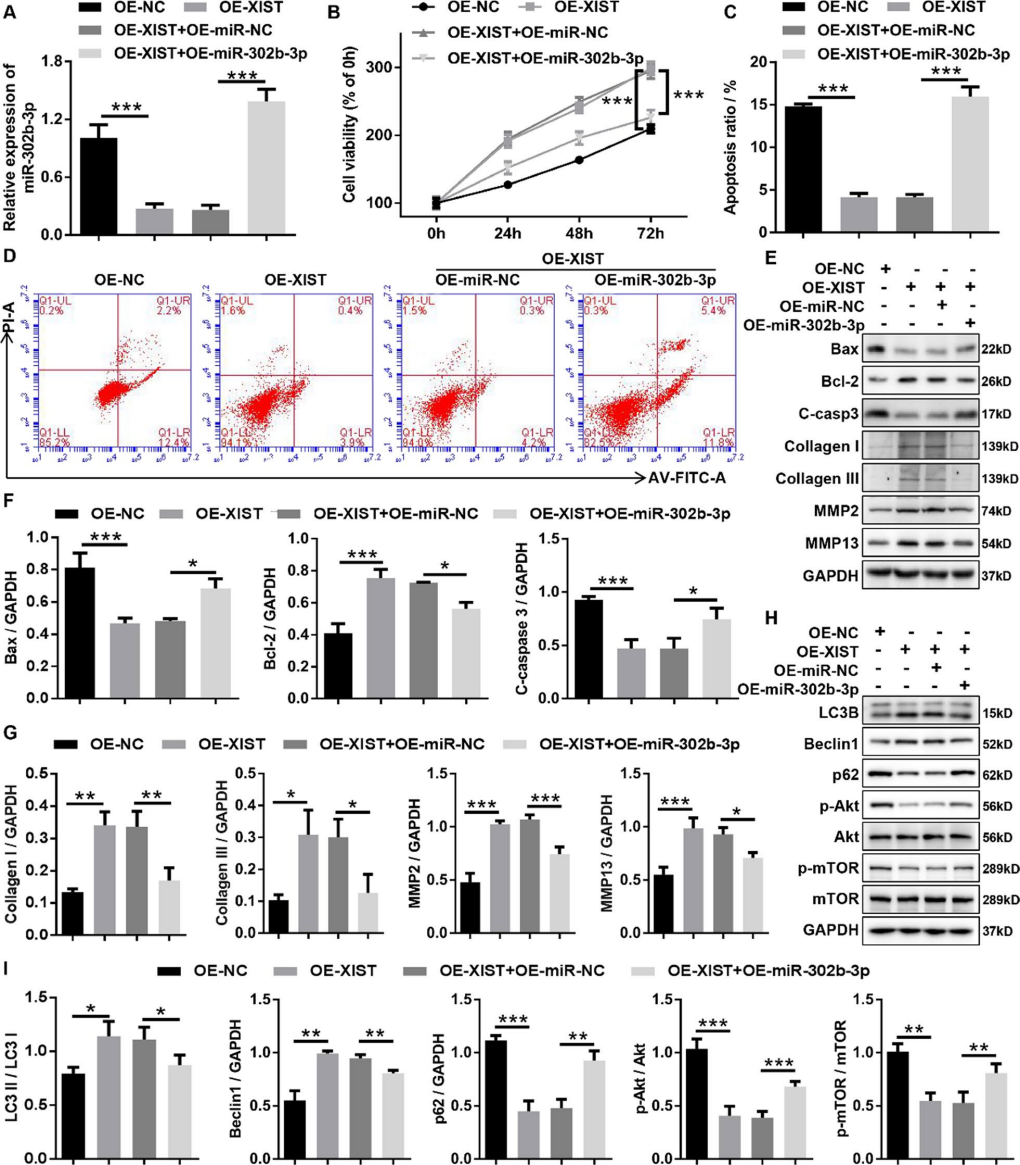
XIST promoted proliferation, fibrosis, and autophagy through regulating the XIST/miR-302b-3p axis in HLF cells. **A** The expression levels of miR-302b-3p in HLF cells infected with the indicated adenovirus were evaluated by RT-qPCR. **B** Growth curves of HLF cells infected with theindicated adenovirus were evaluated by CCK-8 assays. **C** and **D** The apoptosis ratios of HLF cells infected with the indicated adenovirus were assessed by flow cytometry with Annexin V/PI double staining. **E** and **F** The expression of apoptosis-related proteins (cleaved caspase3, Bax, and Bcl-2) in HLF cells infected with the indicated adenovirus was measured by western blotting. **E** and **H** The expression of fibrosis-related proteins (collagen I, collagen III, MMP2, and MMP13) in HLF cells infected with the indicated adenovirus was confirmed by western blotting. **G** and **I** The expression of autophagy-related proteins (LC3B, Beclin1, p62, p-AKT, Akt, p-mTOR, and mTOR) in HLF cells infected with the indicated adenovirus was confirmed by western blotting. C-casp3, cleaved-caspase-3; OE-NC, corresponding negative control of OE-XIST; OE-XIST, the overexpression adenovirus of XIST; OE-miR-NC, corresponding negative control of OE-miR-302b-3p; OE-miR-302b-3p, the overexpression adenovirus of miR-302b-3p. ^*^P < 0.05, ^**^P < 0.01, and.^***^P < 0.001

To explore whether XIST serves its biological function through regulating the XIST/miR-302b-3p/VEGFA axis, rescue experiments were designed using miR-302b-3p overexpression adenovirus (OE-miR-302b-3p). RT-qPCR results displayed that miR-302b-3p overexpression could rescue the inhibitory effect of XIST on miR-302b-3p (Fig. 7A). Growth curves performed by CCK-8 assays indicated that the miR-302b-3p overexpression reversed the proliferation-promoting effects induced by overexpression of XIST in HLF cells (Fig. 7B). At the same times, we found that overexpression of miR-302b-3p significantly rescued the anti-apoptosis of XIST overexpression in HLF cells by flow cytometry analysis with Annexin V/PI double staining and western blotting (Fig. 7C, D, E and F). Moreover, our data displayed that overexpression of miR-302b-3p significantly rescued the fibrosis-promoting effects induced by overexpression of XIST in HLF cells by western blotting (Fig. 7E, G). Meanwhile, we examined whether XIST affected the expression of autophagy-related proteins in HLF cells through miR-302b-3p by western blotting, and found that overexpression of miR-302b-3p significantly rescued the autophagy-activating effects induced by overexpression of XIST in HLF cells through western blot (Fig. 7H, I). In summary, this data uncovered that XIST aggravated HLF cell proliferation, fibrosis, and autophagy through downregulating miR-302b-3p.

## Discussion

Fibrosis is the core pathological feature of HLF development [34]. Hence, it is important to clarify the molecular mechanism of anti-fibrosis and find effective anti-fibrosis targets for the treatment of HLF. With the rapid development of high-throughput sequencing technology and bioinformatics analysis, more and more lncRNAs have been identified in a variety of tissues and cells, which play key regulatory roles in a variety of biological processes [24, 26, 30, 35, 36, 38]. Some of these lncRNAs have been shown to play anti- or pro-fibrotic functions in multiple diseases and have the potential to serve as novel preventive and therapeutic targets for fibrosis [11, 46, 47]. However, lncRNAs associated with HLF have rarely been reported so far. Here, we obtained the expression profiles of lncRNA, miRNA, and mRNA in patients with or without HLF by applying the RNA sequencing. Subsequently, we identified for the first time that XIST was dramatically upregulated in HLF tissues and obviously correlated with LF thinness and fibrosis in patients with LSCS by integrating bioinformatics technology with RT-PCR technology. Many studies have shown that XIST displays vital functions in the regulation of fibrosis [35, 36, 38, 39, 49, 50]. In this study, functional experiments revealed that the overexpression of XIST significantly promoted HLF cells proliferation and fibrosis as well as inhibited apoptosis in vitro, while knockdown of XIST displayed an inhibitory effect on hypertrophy and fibrosis of LF in vivo and in vitro. The biological function of XIST in HLF progression was consistent with its role in other diseases [46, 47, 49, 50]. Zhang et al. reported that XIST increased the proliferation of cardiac fibroblasts and the accumulation of extracellular matrix, leading to myocardial fibrosis. Yang et al. indicated that silencing XIS obviously suppressed renal interstitial fibrosis in diabetic nephropathy. These results uncovered that XIST was a key molecule in HLF progression, and targeted intervention of XIST may be a potential neo-target for HLF treatment.

Autophagy is a highly conserved eukaryotic cell cycle process that plays an core role in maintaining cellular and organismal homeostasis [31]. However, the dysfunction of this process can lead to the development of many diseases, including cancer, cardiovascular disease, arthritis and spinal diseases [14, 28]. A recent study has shown that autophagy may be involved in HLF progression [4]. Our results further confirmed that autophagy levels in HLF tissues was significantly higher than in normal LF tissues, suggesting that autophagy was participated in HLF progression. Increasing evidence has highlighted that overexpression of XIST mediated the enhancement of autophagy activity to regulate the development of multiple diseases [22, 23, 42, 52]. In addition, current studies have shown that autophagy activation in fibroblasts is a sufficient and necessary condition for inducing tissue fibrosis [48]. Hence, western blotting was used to investigate the effect of XIST on autophagy of HLF cells in terms of autophagy-related protein expression levels (LC3II/LC3I, Beclin 1, and p62). The results showed that knockdown of XIST remarkably suppressed autophagy activation by decreasing the LC3II/LC3I ratio and Beclin 1 expression levels as well as increasing p62 expression levels in vitro and in vivo. Further rescue experiments in this study demonstrated that overexpression of XIST promoted proliferation and fibrosis as well as reduced apoptosis in HLF cells by activating autophagy. These findings suggested that autophagy is involved in XIST-induced proliferation and fibrosis of HLF cells, whereas intervention of XIST-mediated autophagy pathway has the potential to become novel therapeutic targets for patients with HLF.

Cumulative evidence has been shown that subcellular localization of lncRNAs can directly determine its mechanism of action, and the cytoplasmic lncRNAs can sequester miRNAs to regulate the miRNA-targeted genes expression [1]. In this study, the nuclear and cytoplasmic RNA fractionation analysis as well as lncRNA FISH indicated that XIST was mainly localized in the cytoplasm of LF cells, suggesting that XIST might promote the pathological progression of HLF by sponging miRNA. More and more research has shown that XIST participates in various diseases progression by sequestering miRNAs to regulate their activity. For example, it has been reported that XIST suppresses myocardial pyroptosis by acting as a sponge for miR-214-3p to disrupt miR-214-3p-mediated Arl2 inhibition [45]. Moreover, XIST acted as a sponge for miR-101 to aggravate cardiac hypertrophy in mice with transverse aortic constriction [41]. Besides, XIST enhanced autophagy of ethanol-induced HSCs through sponging miR-29b to facilitate HMGB1 expression [42]. In our study, our data demonstrated from multiple perspectives that XIST acted as a sponge for miR-302b-3p to exacerbate proliferation, fibrosis, and autophagy in HLF cells. Furthermore, our study also demonstrated that miR-302b-3p was dramatically downregulated in HLF tissues and cells, and overexpression of miR-302b-3p obviously inhibited proliferation, fibrosis, autophagy and promoted apoptosis in HLF cells. Our findings demonstrated that XIST exacerbated HLF development by sponging miR-302b-3p and revealed the significance of XIST/miR-302-3p interaction in LF hypertrophy and fibrosis progression.

According to the ceRNA hypothesis, lncRNAs can act as a ceRNA to modulate miRNA-targeted genes expression. Our results demonstrated that miR-302b-3p could directly interact with VEGFA 3′-UTR by RNA Pull down and dual-luciferase reporter assay. Overexpression of miR-302b-3p led to inhibition of VEGFA levels, whereas silencing miR-302b-3p showed an opposite effect, revealing that VEGFA is a miR-302b-3p target gene in HLF cells. Additionally, we also found that VEGFA was remarkably upregulated in HLF tissue. Consistent with our results, it has been reported that VEGFA is significantly upregulated in HLF tissues [6, 7, 12, 16, 18]. Moreover, a study attested that VEGFA-mediated angiogenesis following mechanical stress may be a critical implementation within HLF pathological progression [10]. VEGFA is known to play a particularly important role in the regulation of tissue fibrosis [25, 35, 36, 38] and autophagy [19, 21, 29]. Emerging studies have uncovered that VEGFA mediated autophagy to regulate the biological functions of various cells, such as granulosa cells [27], cancer cells [19, 21, 37], endothelial cells [33], and so on. In our study, our results demonstrated that miR-302b-3p overexpression inhibited fibrosis and autophagy in HLF cells by downregulating VEGFA expression. To further validate the cross-talk between XIST and VEGFA, our data certified that XIST negatively regulated VEGFA expression in HLF cells, and that this effect could be abolished by miR-302b-3p overexpression or knockdown. These results showed that XIST significantly upregulated VEGFA expression by sponging miR-302b-3p. Similar to our results, other studies have also shown that XIST acts as a ceRNA to indirectly upregulate VEGFA to impair hypoxia-induced angiogenesis [8]. Finally, Pearson correlation analysis displayed that miR-302b-3p expression was negatively correlated with the expression of XIST and VEGFA in the HLF tissue, and VEGFA expression was positively correlated with XIST expression. Taken together, our results would support our hypothesis that XIST functions as a ceRNA to enhance VEGFA-mediated autophagy and fibrosis through decoying miR-302b-3p in HLF progression.

## Conclusions

Our study identified for the first time that lncRNA expression profiles characteristic of HLF in LSS patients, and demonstrated that XIST might act as a ceRNA for miR-302b-3p to regulate VEGFA-mediated autophagy, leading to HLF development. Our findings implied that XIST could be a promising therapeutic target for HLF in the future, and the regulatory network of XIST/miR-302b-3p/VEGFA-mediated autophagy may contribute to a better understand of HLF pathological mechanism.

## Supplementary Information

**Additional file 1**. Related supplementary materials.

## Data Availability

The datasets generated and/or analyzed during the current study available from the corresponding author on reasonable request.
