# Supplementary material for: LncRNA XIST facilitates hypertrophy of ligamentum flavum by activating VEGFA-mediated autophagy through sponging miR-302b-3p
Source: Biol Direct. 2023 May 24;18:25. doi: 10.1186/s13062-023-00383-9 (PMC10207765; doi:10.1186/s13062-023-00383-9)
Supplement: Supplementary file 1 — Additional file 1. Related supplementary materials. [file 13062_2023_383_MOESM1_ESM.docx]

**
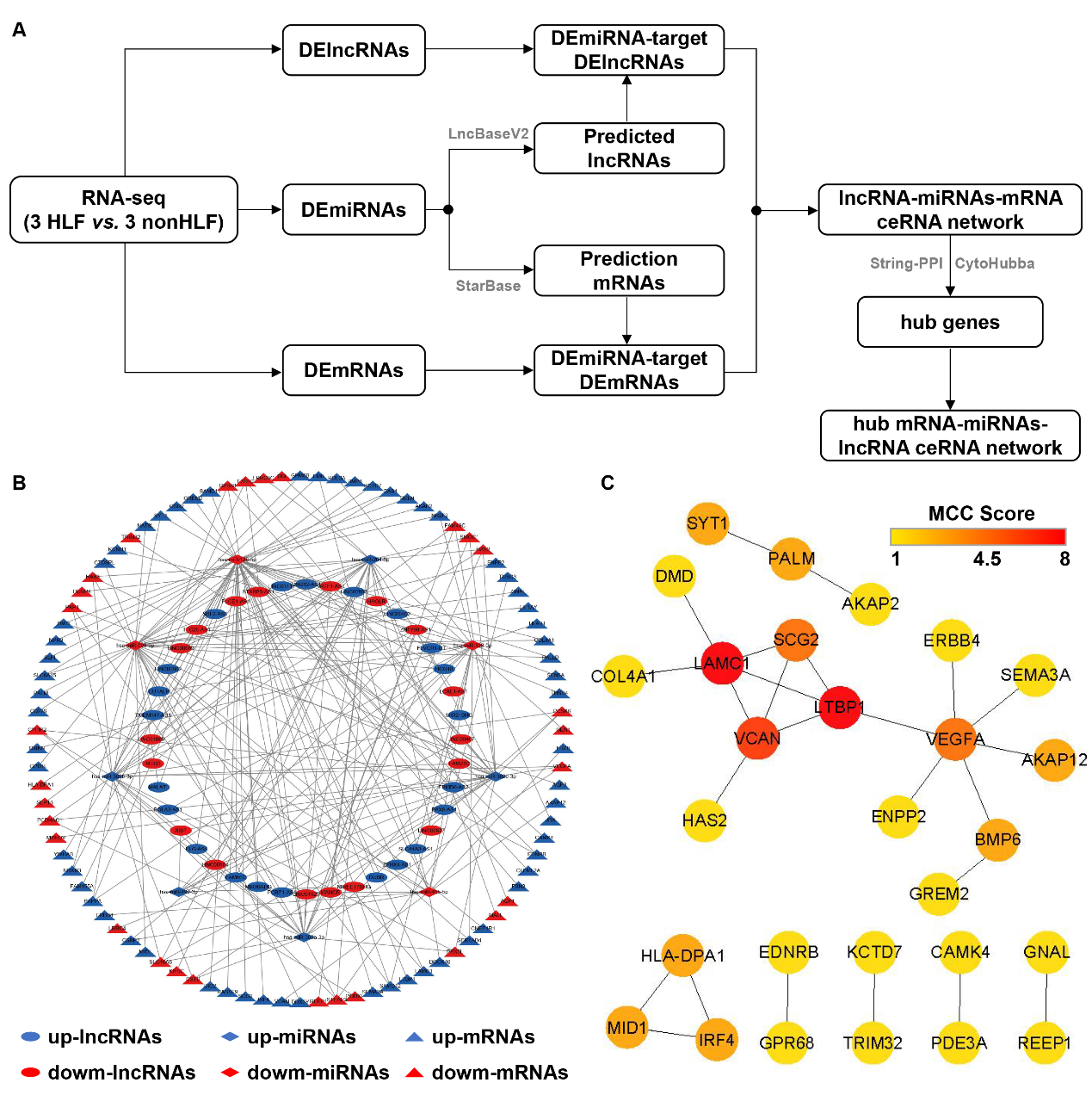
**

**Figure S1 The key target genes in the lncRNAs-miRNAs-mRNAs ceRNA network were identified. (A)** The analysis flow diagram of RNA-seq data. **(B)** Construction of lncRNAs-miRNAs-mRNAs competing endogenous RNA (ceRNA) network in HLF. Ellipses, quadrangle, and triangles represented dysregulated lncRNAs, dysregulated miRNAs, and dysregulated hub mRNAs, respectively. Pink and blue represented upregulated and downregulated RNAs, respectively. **(C)** The hub genes from ceRNA network were identified using cytoHubba.

**
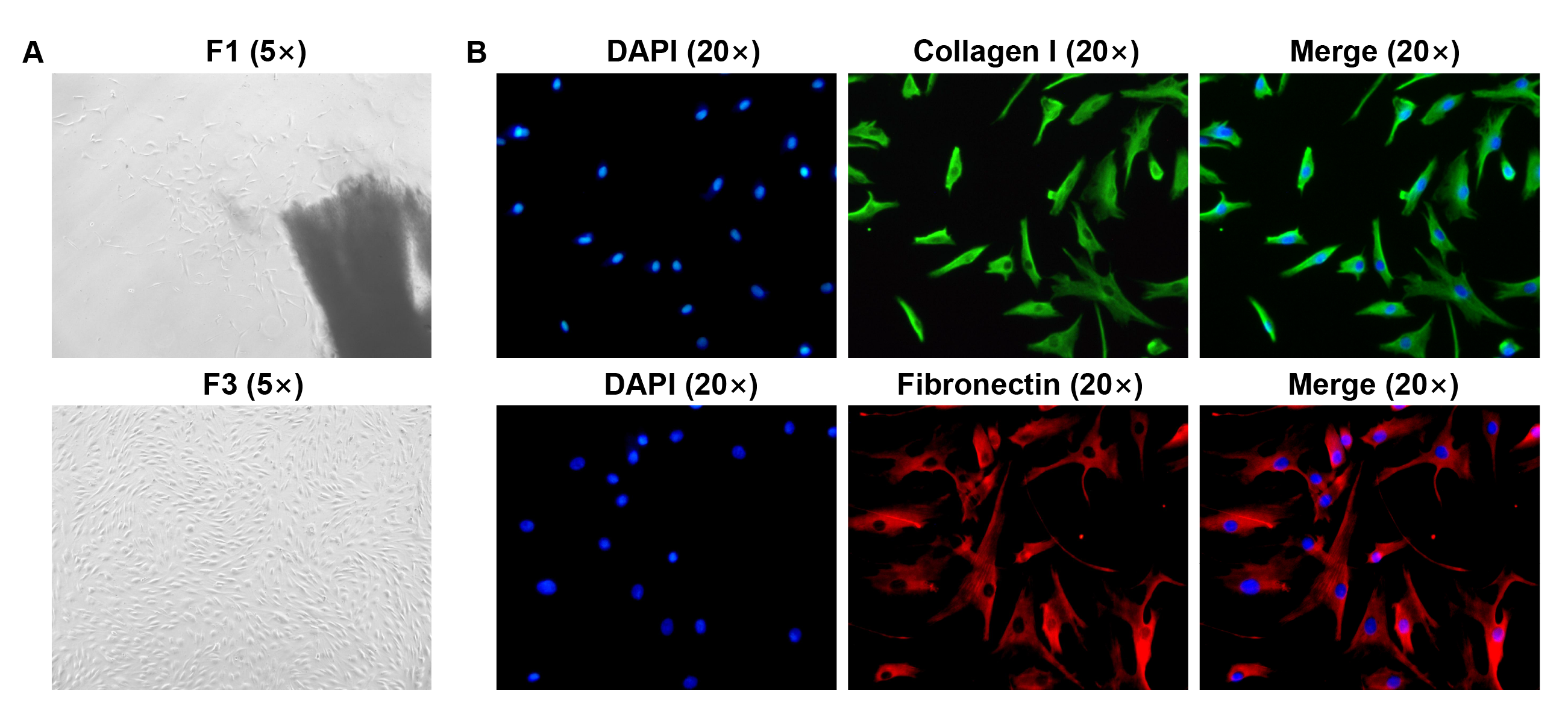
Figure S2 Identify the ligamentum flavum cells. (A)** Pictures showing the morphology of the isolated cells with 1st and 3rd passage from ligamentum flavum tissues. Amplification factor = 5. **(B)** The markers (collagen I and Fibronectin) of ligamentum flavum cells with 3rd passage were detected by immunofluorescence staining. Amplification factor = 20.


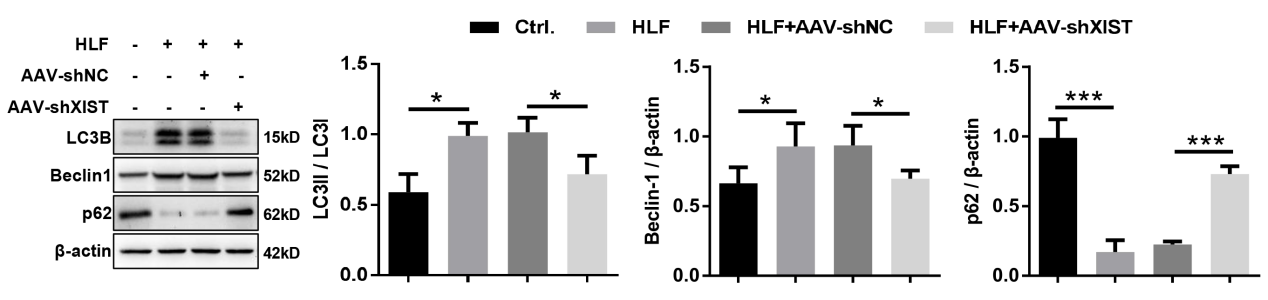


**Figure S3 Knockdown of XIST inhibited autophagy activation in vivo.** The expression levels of autophagy-related proteins (LC3B, Beclin1, and p62) in LF tissue from different treated mice were evaluated by western blotting. Ctrl, control mice; HLF, HLF mice induced by bipedal standing; AAV-shNC, corresponding negative control of AAV-shTCF7; AAV-shTCF7, the RNAi adeno associated virus against XIST. ^*^P < 0.05 and ^***^P < 0.001.


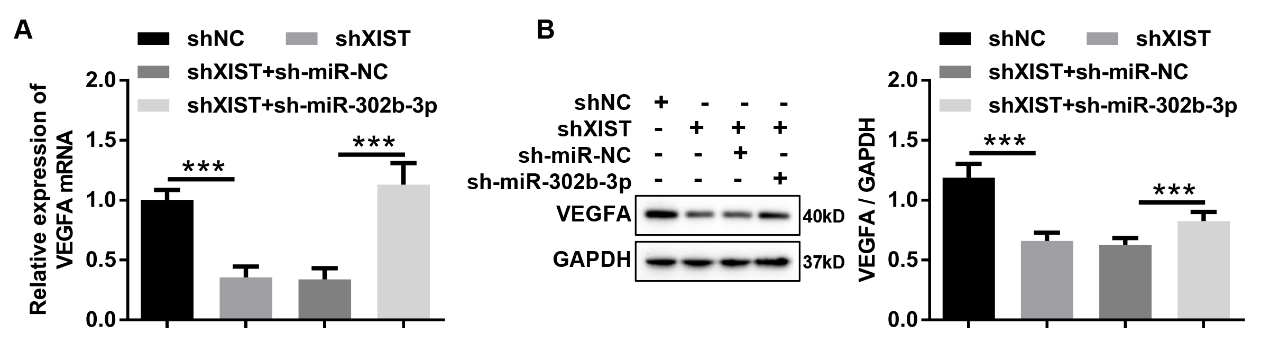


**Figure S4 Knockdown of XIST inhibited VEGFA expression by upregulating miR-302b-3p. (A)** VEGFA mRNA expression in HLF cells infected with the indicated adenovirus was evaluated by RT-qPCR. **(B)** VEGFA protein expression in HLF cells infected with the indicated adenovirus was evaluated by western blotting. shNC, corresponding negative control of shXIST; shXIST, the RNAi adenovirus against XIST; sh-miR-NC, corresponding negative control of sh-miR-302b-3p; sh-miR-302b-3p, the RNAi adenovirus against miR-302b-3p. ^***^P < 0.001.

**Table S1** The information of all enrolled patients in this study.

| **Index** | **LSCS (n = 25)** | **LDH (n = 16)** | **P-values** |
| --- | --- | --- | --- |
| Age | 59±15 | 55±12 | 0.47 |
| Gender | 14 females, 11 males | 8 females, 8 males | 0.58 |
| LF thickness / cm | 0.56±0.06 | 0.25±0.04 | < 0.001 |
| Fibrosis score | 3.16±0.80 | 1.81±0.83 | < 0.001 |
| Lumbar level | L4/5 | L4/5 |  |

**Table S2 The sequences of all the primer in RT-qPCR.**

| **Genes** | **Primer sequences (5’-3’)** |
| --- | --- |
| XIST (human) | Forward, CCCTCATCCCCACTTTTCCC |
|  | Reverse, TGGAATGAGCAGTGTGCGAT |
| XIST (mouse) | Forward, CGTGGCGGTGCAAACTAAAA |
|  | Reverse, ATCAGACGTGTCGTTGGCAT |
| LINC01929 | Forward, GAACCATGGGTTGGCTCTGA |
|  | Reverse, TATGGGCTCCGGGAAAACAC |
| LINC00664 | Forward, GCTCTTCCCCGTCTTAGTGG |
|  | Reverse, ATAACTCCGCCAACCTTCCT |
| NR2F1-AS1 | Forward, AACACGCCCTCAGGTAAAGG |
|  | Reverse, ACATCTGCTGCAACCTGTGA |
| ZNF790-AS1 | Forward, ATTTCCTGACCTCTGACCCG |
|  | Reverse, CCAAATCTATCCACCAGGGC |
| TTC28-AS1 | Forward, CTTGTCTGCTTACGAGGGCT |
|  | Reverse, GGGCGTTGGTTCATGTCTTT |
| LINC00402 | Forward, GGTATTTGGCCCTGCTGGAA |
|  | Reverse, GCCCATCCCCATCATACACA |
| LINC00893 | Forward, CAGAATTCAGGCCTCGTGGT |
|  | Reverse, GGGAGAAGTAGGCGCATCTC |
| TMEM147-AS1 | Forward, CTGAAACAGCCAAGGTGTGC |
|  | Reverse, TCTAGGAGGGTTCATGGCGA |
| LINC00662 | Forward, GCTGGAACCGCCTAAGACAC |
|  | Reverse, AAAGCAGGATTGTAGGGCGG |
| MIR99AHG | Forward, AGCCCAACCTATGTGAAGCC |
|  | Reverse, AGTGGCTGAGGTAGGGATGT |
| MALAT1 | Forward, GCTCTGTGGTGTGGGATTGA |
|  | Reverse, GTGGCAAAATGGCGGACTTT |
| VEGFA | Forward, ATAAGTCCTGGAGCGTTCCCT |
|  | Reverse, GGTGAGAGATCTGGTTCCCG |
| GAPDH | Forward, GCGGGACAACTACGGGAAGAAG |
|  | Reverse, AGCACTGTCATCGGAAGGAACG |
| miR-302a-3p | RT, GTCGTATCCAGTGCGTGTCGTGGAGTCGGCAATTG  CACTGGATACGACTCACCAA |
|  | Forward, CGCTAAGTGCTTCCATGTT |
|  | Reverse, GTCGTATCCAGTGCGTGTC |
| miR-302b-3p | RT, GTCGTATCCAGTGCGTGTCGTGGAGTCGGCAATTG  CACTGGATACGACCTACTAA |
|  | Forward, CGTAAGTGCTTCCATGTT |
|  | Reverse, GTCGTATCCAGTGCGTGTC |
| miR-302d-3p | RT, GTCGTATCCAGTGCGTGTCGTGGAGTCGGCAATTG  CACTGGATACGACACACTCAA |
|  | Forward, CGCUAAGTGCTTCCATGT |
|  | Reverse, GTCGTATCCAGTGCGTGTC |
| U6 | Forward, CTCGCTTCGGCAGCACATATACT |
|  | Reverse, CAGTGCGTGTCGTGGAGT |

**Table S3 The sequences of shRNAs or miR-302b-3p inhibitor/mimics were used in this study.**

| Names | Sequences (5’-3’) |
| --- | --- |
| shXIST (human) | GCTATAAGAGGCTCCAAATTA |
| shXIST (mouse) | CTCGGATACCTGCTGTTATTA |
| shVEGFA (human) | AGGGCAGAATCATCACGAAGT |
| miR-302b-3p mimics (human) | sense, UAAGUGCUUCCAUGUUUUAGUAG  antisense, ACUAAAACAUGGAAGCACUUAUU |
| NC mimics (human) | UUGUACUACACAAAAGUACUG |
| miR-302b-3p inhibitor (human) | CUACUAAAACAUGGAAGCACUUA |
| NC inhibitor (human) | CAGUACUUUUGUGUAGUACAA |
